# Thyroid cancer incidence in the female population of Georgia by regions and municipalities

**DOI:** 10.1093/eurpub/ckac131.015

**Published:** 2022-10-25

**Authors:** N Abesadze, V Tkeshelashvili, M Kereselidze, R Kvanchakhadze, T Beruchashvili

**Affiliations:** School of Health Sciences, University of Georgia, Tbilisi, Georgia

## Abstract

**Background:**

According to the NCDC (Tbilisi, Georgia), in 2015-2019, thyroid cancer ranked second in the cancer structure of cancer in Georgia. Additional studies are needed to identify the thyroid cancer incidence by regions and municipalities of Georgia.

**Methods:**

E-dBase of cancer population registry for 2015-2019 (52,178 cancer cases), Tbilisi registry database for 2002-2004 (33,478 deaths from all causes), methodology recommended by IARC (Lyon) and UICC (Geneva), SEER program and 2014 Georgian census data were used in the study. Standardized cancer incidence and mortality rates (ASR, TASR, AAR, SRR, CR64, CR74, SIR, SMR, PIR, 95% CI) were calculated.

**Results:**

Ranking and proportion of thyroid cancer in female population of Georgia in 2015-2019 according to the regions and municipalities, its age specifics and dynamics were determined. Incidence of thyroid cancer in women in Tbilisi (ASR=52.4%000; AAR=64.1%000), compared to Georgia (ASR=34.4%000; AAR=41.0%000), indicates that Tbilisi is the geographically highest prevalence zone for this site cancer and the highest levels were observed in the 25-69 age group (TASR25-69 - Georgia = 110.8%000, Tbilisi = 190.1%000). In dynamics, the incidence of thyroid cancer in the 27-year period (2015-2019 vs 1988-1992) according to the SIR, increased by 66.4%. According to the cumulative risk index (CR64, CR74), the municipalities, where the risk of developing thyroid cancer is almost 1.5 times higher than the total rate in Georgia, were identified. According to the PIR, the ratio of thyroid cancer to the share of thyroid cancer in the structure of cancer in the regions of Georgia (including Tbilisi) showed that the proportion of thyroid cancer in Tbilisi (PIR=117.7) is 17.7% higher compared to proportion of total thyroid cancer in Georgia.

**Conclusions:**

It is recommended that the epidemiological map of thyroid cancer incidence be used in planning national, regional, and municipal preventive programs.

**Key messages:**

• It is recommended to continue study in this direction: retrospective review of histological and histochemical features of each case of thyroid cancer.

• It is recommended: to conduct molecular (oncogenes) studies in conjunction with histological and histochemical studies.

